# Pulse pressure variation and stroke volume variation under different inhaled concentrations of isoflurane, sevoflurane and desflurane in pigs undergoing hemorrhage

**DOI:** 10.6061/clinics/2015(12)07

**Published:** 2015-12

**Authors:** Alexandre Hideaki Oshiro, Denise Aya Otsuki, Marcelo Waldir M Hamaji, Kaleizu T Rosa, Keila Kazue Ida, Denise T Fantoni, José Otavio Costa Auler

**Affiliations:** I Faculdade de Medicina da Universidade de São Paulo, Programa de Pós-Graduação em Anestesiologia, São Paulo/SP, Brazil.; II Faculdade de Medicina da Universidade de São Paulo, Laboratório de Investigação Médica (LIM-08/ Anestesiologia), São Paulo/SP, Brazil.; III Faculdade de Medicina Veterinária e Zootecnia da Universidade de São Paulo, Departamento de Cirurgia, São Paulo/SP, Brazil.

**Keywords:** Pulse Pressure Variation, Stroke Volume Variation, Inhalant Anesthetics

## Abstract

**OBJECTIVES:**

Inhalant anesthesia induces dose-dependent cardiovascular depression, but whether fluid responsiveness is differentially influenced by the inhalant agent and plasma volemia remains unknown. The aim of this study was to compare the effects of isoflurane, sevoflurane and desflurane on pulse pressure variation and stroke volume variation in pigs undergoing hemorrhage.

**METHODS:**

Twenty-five pigs were randomly anesthetized with isoflurane, sevoflurane or desflurane. Hemodynamic and echocardiographic data were registered sequentially at minimum alveolar concentrations of 1.00 (M1), 1.25 (M2), and 1.00 (M3). Then, following withdrawal of 30% of the estimated blood volume, these data were registered at a minimum alveolar concentrations of 1.00 (M4) and 1.25 (M5).

**RESULTS:**

The minimum alveolar concentration increase from 1.00 to 1.25 (M2) decreased the cardiac index and increased the central venous pressure, but only modest changes in mean arterial pressure, pulse pressure variation and stroke volume variation were observed in all groups from M1 to M2. A significant decrease in mean arterial pressure was only observed with desflurane. Following blood loss (M4), pulse pressure variation, stroke volume variation and central venous pressure increased (*p*<0.001) and mean arterial pressure decreased in all groups. Under hypovolemia, the cardiac index decreased with the increase of anesthesia depth in a similar manner in all groups.

**CONCLUSION:**

The effects of desflurane, sevoflurane and isoflurane on pulse pressure variation and stroke volume variation were not different during normovolemia or hypovolemia.

## INTRODUCTION

Pulse pressure variation (PPV) and stroke volume variation (SVV) are dynamic parameters of preload used to predict fluid responsiveness in mechanically ventilated patients. These indicators have been used in different experimental models, such as sepsis, acute lung injury, hemodilution and hemorrhage, and during the administration of various vasoactive drugs 1,6. These indicators have also been studied in clinical settings, including major surgeries, sepsis and pulmonary hypertension. PPV and SVV have been compared to each other and compared to other hemodynamic indicators to assess fluid responsiveness 7-10. The limitations of the use of these parameters have been described and include the requirement for mechanical ventilation, fixed tidal volumes and a sinus rhythm, among others 11,12.

The effects of the commonly used inhalational anesthetics isoflurane, desflurane and sevoflurane are well described in the medical literature. Each of these anesthetics can cause dose-dependent depression in myocardial contractility 13,14 and vasodilation of the peripheral vascular bed, leading to a decrease in cardiac index (CI) and systemic arterial pressure 15-19.

The use of different anesthetic agents in the operating room necessitates an investigation of whether they differentially influence PPV and SVV and whether equipotent doses of different anesthetics elicit equivalent responses in different hemodynamic conditions. To our knowledge, no previous study has evaluated this issue. The purpose of this study was to assess the effects of three inhaled agents with equipotent doses on PPV and SVV during normovolemia and hypovolemia.

## METHODS

This study was approved by the Ethics Committee for Review of Research Projects (CAPPesq 0697/08) at our institution. Twenty-five young, healthy Large White Landrace pigs of both genders weighing 28.2±3.0 kg were used in this study. The animals were fasted overnight and their water supply was interrupted three hours before the procedure.

### Anesthesia and Preparation

Anesthesia was induced in all animals via a mask with isoflurane, sevoflurane or desflurane (according to prior randomization) in 100% oxygen. After orotracheal intubation with a cuffed endotracheal tube, the fraction of inspired oxygen (FiO_2_) was decreased to 40% and maintained at that level throughout the procedure. The lungs were ventilated (Primus, Draeger Medical, Lubeck, Germany) using the volume-controlled mode, with a tidal volume (Vt) of 8 mL/kg, an inspiratory:expiratory ratio of 1:2, a positive end-expiratory pressure of 5 cmH_2_O and a respiratory rate (20-23 breaths/min) that was adjusted to maintain the partial pressure of carbon dioxide in the arterial blood (PaCO_2_) at 35-40 mmHg. Pulse oximetry was measured with a sensor placed on the tongue of the animal and connected to a multiparameter monitor (IntelliVue MP40, Philips Medical Systems, Andover, USA). Lactated ringer’s solution was administered intravenously throughout the experiment via a catheter (Abbocath Tplus, Illinois USA) introduced in the auricular vein at an infusion rate of 5 mL/kg/h. This vein was also used to administer pancuronium bromide at an infusion rate of 0.3 mg/kg/h after the minimum alveolar concentrations (MACs) were determined.

A 7.5F pulmonary artery catheter (Edwards CCO catheter connected to an Edwards Vigilance CCO Monitor; Edwards Lifesciences Corp., Irvine, CA, USA) was inserted into the right external jugular vein to measure cardiac output (CO) using the thermodilution technique. Adequate positioning of the pulmonary artery catheter was confirmed by the observation of typical waveforms. The right femoral artery was surgically exposed and catheterized for arterial pressure measurement and blood collection. Heart rate (HR) and rhythm, mean arterial pressure (MAP), central venous pressure (CVP), pulmonary artery occlusion pressure (PAOP) and SVV were recorded directly from a multiparameter monitor (IntelliVue MP40, Philips Medical Systems, Andover, USA). Automated PPV was obtained from another multiparameter monitor (DX2020, Dixtal, São Paulo, Brazil). Temperature was maintained within the normal limits for piglets (38°C) using warm blankets (Medi-Therm II, Gaymar Industries, Orchard Park, NY, USA). Transesophageal echocardiography (TEE) was performed using a 7.5/5.0 MHz TEE transducer (Omniplane III – Philips Medical, Andover, MA, USA) connected to an ultrasound system (EnVisor Performance, Philips Medical, Andover, MA, USA). Systolic function was determined using a modified version of Simpson's method. Left ventricular diastolic function was measured using Doppler patterns of mitral inflow. Isovolumic relaxation time (IVRT) was measured as the time from aortic valve closure (determined based on the sub-aortic valve pulsed Doppler flow profile) to the onset of transmitral inflow. All echocardiographic measurements were performed according to the American Society of Echocardiography recommendations 20.

### Minimun Alveolar Concentration Determination

The MAC was determined using the method described by Quasha et al. 21 and Eger et al. 22. Fifteen minutes was allowed for anesthetic stabilization, during which time each inhalant agent was provided at the end-tidal concentration equivalent to 1 MAC for young pigs: 3.5% for sevoflurane 23; 10% for desflurane; and 2% for isoflurane 22. After the equilibration period, a hemostat was clamped to a full ratchet lock on the proximal phalanges of the 3^rd^ or 4^th^ digit for 60 seconds. In the presence of pedal withdrawal or gross and purposeful movements of the legs and/or head, the end-tidal concentration of the volatile anesthetic was increased to 110% of the actual expired concentration; in the absence of purposeful movements in response to the stimulus, the end-tidal concentration was decreased to 90% of the end-tidal concentration. Thereafter, another 15 minutes was allowed for anesthetic stabilization and the protocol was repeated until the end-tidal concentration of the inhalant agent had decreased to a level that caused the response to the stimuli to be between a positive and negative response; this end-tidal concentration was defined as the MAC for that agent and was determined for each animal individually. The stimulation site was altered slightly between stimulations to prevent sensitization and desensitization to subsequent stimuli.

### Experimental Protocol

At the beginning of the experiment, the animals were randomly assigned to one of the three groups: isoflurane (n=8, ISO), sevoflurane (n=8, SEVO) or desflurane (n=9, DESF). Baseline parameters were recorded following instrumentation and under steady-state anesthesia with 1 MAC (M1). The end-tidal concentration of the inhalant agent was then increased to 1.25 MAC for 20 minutes (M2) and decreased again to 1 MAC for 20 minutes (M3). Thereafter, at 1 MAC, hypovolemia was induced by withdrawing 30% of the estimated blood volume and after a 20-minute stabilization period (M4), the end-tidal concentration of the anesthetic was increased to 1.25 MAC (M5). Each step of the experiment lasted 20 minutes and was followed by hemodynamic, echocardiographic, PPV and SVV data collection.

### Statistical Analysis

All values are shown as the mean±standard deviation (SD). The data were tested for normality using the Kolmogorov-Smirnov test. Parametric data were compared within groups and between groups by analysis of variance (ANOVA) for repeated measures with the Tukey-Kramer post-hoc multiple comparison test. Data that were abnormally distributed were analyzed within groups using the Friedman test and between groups using the Kruskal-Wallis test. When appropriate, a post-hoc multiple comparison analysis was performed using Dunn’s test (SigmaStat 3.11, Systat Software Inc., San Jose, CA, USA). Differences with *p*-values <0.05 were considered statistically significant.

## RESULTS

The mean values for 1 MAC were 14.1±1.6% for desflurane, 4.1±0.6% for sevoflurane and 2.6±0.3% for isoflurane. Mean total blood volumes of 681±70 mL, 643±65 mL and 623±62 mL were withdrawn from the DESF, SEVO and ISO groups, respectively.

### Pulse Pressure Variation and Stroke Volume Variation

**Figure 1:**
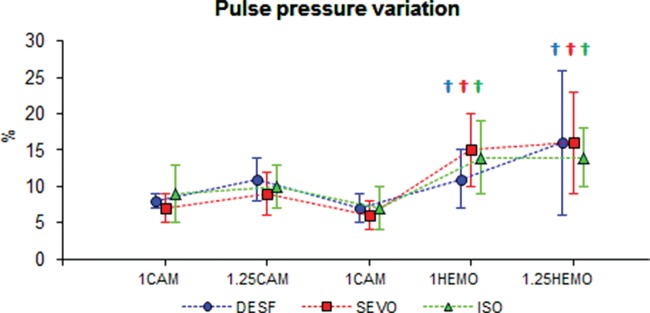
Pulse pressure variation (PPV) (mean ± SD) for animals anesthetized with desflurane (DESF), sevoflurane (SEVO) or isoflurane (ISO). †: *p*<0.05 different from M3.

No significant changes in PPV or SVV were observed when the MAC was increased from 1 (M1) to 1.25 (M2) or when it was then decreased from 1.25 to 1 (M3) (Figure 1). After blood withdrawal (M4), PPV and SVV increased significantly compared to at M3. When the MAC was increased to 1.25 MAC during the hypovolemic state (M5), PPV and SVV were significantly greater than the values at M3, but not those at M4. Throughout the entire procedure, no significant differences in PPV or SVV were observed between the DESF, SEVO and ISO groups.

### Hemodynamic Parameters

No significant hemodynamic differences between groups were observed at baseline (1 MAC) (Table 1). The increase in MAC to 1.25 MAC was associated with a decrease in CI and an increase in CVP in all animals. Although the MAP decreased in all groups from M1 to M2, this decrease was only significant in the DESF group (*p*<0.001). After the MAC was returned to 1 (M3), all hemodynamic parameters returned to their baseline values.

Blood withdrawal was followed by a significant decrease in MAP, CVP, MPAP and PAOP. In the ISO group, a significant increase in HR was observed at M4 compared with baseline (*p*<0.05). The MAC increase under hypovolemia (M5) caused different effects on MAP and HR between groups. In the DESF and ISO groups, MAP decreased significantly from M4 to M5 (*p*<0.001 and *p*<0.001, respectively). At M5, HR was lower in the DESF group than in the ISO and SEVO groups (*p*<0.001). The MAC increase to 1.25 MAC (M5) resulted in an increase in HR in the SEVO group and a decrease in HR in the ISO group. However, both of these groups showed a higher HR than the DESF group (*p*<0.05). The MAC increase at M5 also resulted in a decrease in CI in all groups compared to the CI values at M3 and M4 (*p*<0.001).

The echocardiographic parameters (systolic and diastolic functions) were not affected by the changes in the MAC values (Table 2). End-diastolic volume (EDV) was lower in all groups after hemorrhage compared to at M3. Additionally, the MAC increase after blood withdrawal (M5) resulted in an increase in EDV compared to its value at M3 in all groups.

## DISCUSSION

The present study shows that desflurane, sevoflurane and isoflurane, at 1 and 1.25 MAC, have similar effects on PPV and SVV in pigs under normovolemia and hypovolemia. Despite the hemodynamic alterations caused by the increase in anesthesia depth and hemorrhage, only hemorrhage resulted in a notable increase in cardiac responsiveness.

Similarly, the increase in anesthesia depth with desflurane, sevoflurane and isoflurane induced a slight increase in PPV and SVV. This finding was attributed to the dose-dependent myocardial depression caused by the volatile agents, which resulted in decreases in CI of 20%, 23% and 15% in the desflurane, sevoflurane and isoflurane groups, respectively. This cardiac depression caused a modest decrease in arterial pressure (10% and 8% with sevoflurane and isoflurane, respectively) that was more prominent with desflurane (19%). A reduction in arterial pressure was induced by desflurane (9%), but not with sevoflurane (increase of 10%) or isoflurane (increase of 5%) and this result was attributed to the decrease in systemic vascular resistance induced by that anesthetic. A decrease in arterial pressure can cause relative hypovolemia, which is reflected by increases in PPV and SVV 3. Despite the hypotension caused by desflurane, it did not impact PPV or SVV differently from sevoflurane or isoflurane, indicating that these indexes are better indicators of cardiac responsiveness than volemia. In addition, the slight increase in PPV caused by increasing the MAC was similar for all three anesthetics and did not reach the estimated threshold value of 13% previously described to discriminate between responders and non-responders to fluid loading 24.

The blood loss induced a significant decrease in arterial pressure in all groups, initially (M4) due to the decrease in SVR and later, also due to the decrease in CI (M5). The CI was initially preserved in all groups, as the HR compensated for the blood loss. Thereafter, CI markedly decreased with the increase in the MAC with all three anesthetics. Hypovolemia decreased the left ventricular preload in all groups and this resulted in an increase in PPV and SVV that was not further increased by increasing the anesthesia depth. This finding suggests that myocardial function was preserved, with the ventricles operating on the ascending portion of the Frank-Starling curve, indicating an increase in cardiac responsiveness under hypovolemia 25,26.

The higher MAC values for desflurane, sevoflurane and isoflurane obtained in this study compared to those reported in the literature 22,23 may be attributed to several factors, including differences in age, body temperature, employment of pre-anesthetic and induction agents and, most importantly, the type and site of the noxious stimulus 21,22. In a previous study that employed animals of the same origin, breed and age, the sevoflurane MAC was also higher than those reported in the literature and was similar to that obtained in this study 27. Moreover, in pilot studies using 18% end-tidal desflurane, we observed MAP values below 40 mmHg and death immediately after a 30% blood volume hemorrhage. Lower end-tidal concentrations were not employed due to ethical concerns associated with the use of neuromuscular blockers during a light plane of anesthesia.

This experimental study had some limitations, including the absence of a fluid challenge. The goal of this study was to evaluate whether a decrease in preload caused by blood withdrawal would be reflected by changes in PPV and SVV, not to demonstrate the efficacy of these dynamic parameters. Additionally, volume changes after a fluid challenge performed at 1 MAC would affect the values measured at the next time-point, even if a small fluid volume was employed. Another limitation of this study was the use of 1 and 1.25 MAC. It is possible that higher end-tidal volumes (e.g., 1.5 MAC) could have amplified the hemodynamic alterations observed, but the high desflurane end-tidal volumes (14.1±1.6%) at 1 MAC made it impossible to achieve 1.5 MAC (≈21%), as the maximal concentration provided by the vaporizer was 18%.

In conclusion, desflurane, sevoflurane and isoflurane do not cause significant changes in the functional preload parameters of PPV or SVV when the MAC is increased from 1 to 1.25 in normovolemic and hypovolemic pigs.

## ACKNOWLEDGMENTS

This work was supported by grants from FAPESP (2008-57247-0 and 2008-57248-6), CAPES/PROAP and FMUSP/LIM.

## Figures and Tables

**Table 1:** Hemodynamic changes in pigs anesthetized with desflurane, sevoflurane and isoflurane while undergoing hemorrhage (mean ± standard deviation).

Parameter	Groups	Time-points				
					Withdrawal of 30% of the estimated blood volume	
		M1 (1.00 MAC)	M2 (1.25 MAC)	M3 (1.00 MAC)	M4 (1.00 MAC)	M5 (1.25 MAC)
HR (bpm)	DESF	95±8	97±7	100±8	110±10	98±24
	SEVO	96±18	97±18	98±19	109±29	125±41*†d
	ISO	109±17	106±12	116±10	144±32*†s,d	126±17^d^
MAP (mmHg)	DESF	62±6	50±8^*^	65±6	51±9†	38±11†‡
	SEVO	68±13	61±7	69±10	52±5†	57±12†d
	ISO	65±9	60±7	73±7	57±11†	50±10†d
MPAP (mmHg)	DESF	19±1	19±1	21±2^*^	17±3†	16±2†
	SEVO	19±2	19±2	22±4^*^	16±2†	17±3†
	ISO	20±1	21±2	21±2^*^	17±2†	19±3†
PAOP (mmHg)	DESF	13±1	13±1	14±1	8±1†	9±2†‡
	SEVO	14±2	14±2	14±1	8±2†	10±3†‡
	ISO	12±2	14±3	13±3	8±2†	10±3†‡
CVP (mmHg)	DESF	10±1	11±1^*^	11±1	6±1†	7±1†‡
	SEVO	9±2	10±2^*^	10±2	6±2†	7±2†‡
	ISO	10±2	11±2^*^	10±2	7±2†	8±2†‡
CI (L.min^−1^.m^−2^)	DESF	3.6±0.6	2.9±0.5^*^	3.8±0.5	3.7±0.7	2.6±1.0†‡
	SEVO	4.0±0.1	3.1±0.4^*^	3.9±0.6	3.1±0.5	3.3±0.7†‡
	ISO	4.2±0.1	3.6±0.9^*^	4.5±1.3	4.1±0.9	3.5±0.7†‡
SVRI (dynes.s.cm^−5^.m^−2^)	DESF	1169±171	1064±143	1164±104	1017±236†	1004±114†
	SEVO	1200±86	1331±155	1228±186	1214±218†	1221±119†
	ISO	1047±145	1096±187	1182±236	996±157†	928±128†
PVRI (dynes.s.cm^−5^.m^−2^)	DESF	146±43	192±59*s	156±33	197±65	226±112†‡
	SEVO	118±33	136±25	138±19	201±36†	186±33
	ISO	140±27	143±24	155±28	183±31	201±33
SVV	DESF	9±3	12±5	10±4	14±8†	14±4†
	SEVO	9±6	10±6	11±2	14±3†	14±7†
	ISO	6±1	10±5	9±6	12±2†	13±4†

DESF: desflurane group; SEVO: sevoflurane group; ISO: isoflurane group; HR: heart rate; MAP: mean arterial pressure; MPAP: mean pulmonary artery pressure; PAOP: pulmonary artery occlusion pressure; CVP: central venous pressure; CI: cardiac index; SVRI: systemic vascular resistance index; PVRI: pulmonary vascular resistance index; SVV: stroke volume variation. *: *p*<0.05 different from M1. †: *p*<0.05 different from M3; ‡: *p*<0.05 different from M4; d: *p*<0.05 different from DESF group; s: *p*<0.05 different from SEVO group.

**Table 2:** Echocardiographic changes in pigs anesthetized with desflurane, sevoflurane and isoflurane while undergoing hemorrhage (mean ± standard deviation).

Parameters	Groups	Time-points				
					Withdrawal of 30% of the estimated blood volume	
		M1 (1.00 MAC)	M2 (1.25 MAC)	M3 (1.00 MAC)	M4 (1.00 MAC)	M5 (1.25 MAC)
EDV (mL)	DESF	50.0±4.2	54.7±13.3	47.0±5.3	43.6±6.1†	39.5±5.0†
	SEVO	48.2±11.5	56.0±12.3	42.9±17.3	33.2±9.3†	32.6±10.2†
	ISO	37.3±6.0	44.7±6.0	43.1±3.4	31.3±8.5†	24.9±6.5†
ESV (mL)	DESF	22.9±3.7	23.2±3.4	26.2±6.5	23.3±6.5	23.5±5.5
	SEVO	26.1±8.9	28.3±5.4	22.3±7.7	16.9±2.1	15.5±5.3
	ISO	19.6±2.2	20.3±2.1	20.2±5.0	15.1±6.5	11.5±3.9†d
EF (%)	DESF	54.3±4.7	55.1±13.8	43.7±9.5	48.9±8.1	41.1±7.9
	SEVO	46.7±5.2	48.8±8.4	46.2±10.5	47.5±11.4	52.7±11.8
	ISO	46.2±10.7	52.8±12.8	52.5±13.7	53.2±15.1	54.5±4.9
LVEDA	DESF	17.4±0.3	18.1±1.9	17.8±1.4	16.5±1.5	14.4±2.2†‡
	SEVO	17.9±3.6	19.7±3.0	16.1±4.2	14.4±2.9	14.2±2.6†‡
	ISO	14.6±1.5	15.8±0.9	15.9±1.5	13.4±2.5	11.5±2.4†‡
E/A ratio	DESF	2.0±0.7	2.0±1.0	1.7±0.2	1.8±0.4	1.7±0.6
	SEVO	1.9±0.5	1.5±0.4	1.5±0.5	1.2±0.6	1.6±0.4
	ISO	1.5±0.5	1.9±0.4	1.8±0.3	1.5±0.6	2.0±0.5
IVRT (mseg)	DESF	75±12	84±16	81±17	85±15	74±19
	SEVO	67±8	90±19	82±20	76±18	94±5
	ISO	101±36s	78±17	86±17	56±7†d	65±20s

EDV: end-diastolic volume; ESV: end-systolic volume; EF: ejection fraction; LVEDA: left ventricle end-diastolic area; IVRT: isovolumic relaxation time; *: *p*<0.05 different from M1. †: *p*<0.05 different from M3; ‡: *p*<0.05 different from M4; d: *p*<0.05 different from DESF group; s: *p*<0.05 different from SEVO group.

## References

[1] da Silva Ramos FJ, de Oliveira EM, Park M, Schettino GP, Azevedo LC (2011). Heart-lung interactions with different ventilatory settings during acute lung injury and hypovolaemia: an experimental study. Br J Anaesth.

[2] de Oliveira MA, Otsuki DA, Noel-Morgan J, Leite VF, Fantoni DT, Auler JO (2009). A comparison between pulse pressure variation and right end diastolic volume index as guides to resuscitation in a model of hemorrhagic shock in pigs. J Trauma.

[3] Westphal GA, Goncalves AR, Bedin A, Steglich RB, Silva E, Poli-de-Figueiredo LF (2010). Vasodilation increases pulse pressure variation, mimicking hypovolemic status in rabbits. Clinics.

[4] Wajima Z, Shiga T, Imanaga K, Inoue T (2012). Do induced hypertension and hypotension affect stroke volume variation in man. J Clin Anesth.

[5] Daudel F, Tuller D, Krahenbuhl S, Jakob SM, Takala J (2010). Pulse pressure variation and volume responsiveness during acutely increased pulmonary artery pressure: an experimental study. Crit Care.

[6] Sant'Ana AJ, Otsuki DA, Noel-Morgan J, Leite VF, Fantoni DT, Abrahao Hajjar L (2012). Use of pulse pressure variation to estimate changes in preload during experimental acute normovolemic hemodilution. Minerva Anestesiol.

[7] Michard F, Boussat S, Chemla D, Anguel N, Mercat A, Lecarpentier Y (2000). Relation between respiratory changes in arterial pulse pressure and fluid responsiveness in septic patients with acute circulatory failure. Am J Respir Crit Care Med.

[8] Auler JO, Galas F, Hajjar L, Santos L, Carvalho T, Michard F (2008). Online monitoring of pulse pressure variation to guide fluid therapy after cardiac surgery. Anesth Analg.

[9] Wyler von Ballmoos M, Takala J, Roeck M, Porta F, Tueller D, Ganter CC (2010). Pulse-pressure variation and hemodynamic response in patients with elevated pulmonary artery pressure: a clinical study. Crit Care.

[10] Lopes MR, Auler JO, Michard F (2006). Volume management in critically ill patients: New insights. Clinics.

[11] Mesquida J, Kim HK, Pinsky MR (2011). Effect of tidal volume, intrathoracic pressure, and cardiac contractility on variations in pulse pressure, stroke volume, and intrathoracic blood volume. Intensive Care Med.

[12] Kim HK, Pinsky MR (2008). Effect of tidal volume, sampling duration, and cardiac contractility on pulse pressure and stroke volume variation during positive-pressure ventilation. Crit Care Med.

[13] Pagel PS, Kampine JP, Schmeling WT, Warltier DC (1991). Influence of volatile anesthetics on myocardial contractility in vivo: desflurane versus isoflurane. Anesthesiology.

[14] Boban M, Stowe DF, Buljubasic N, Kampine JP, Bosnjak ZJ (1992). Direct comparative effects of isoflurane and desflurane in isolated guinea pig hearts. Anesthesiology.

[15] Park WK, Pancrazio JJ, Suh CK, Lynch C (1996). Myocardial depressant effects of sevoflurane. Mechanical and electrophysiologic actions in vitro. Anesthesiology.

[16] Harkin CP, Pagel PS, Kersten JR, Hettrick DA, Warltier DC (1994). Direct negative inotropic and lusitropic effects of sevoflurane. Anesthesiology.

[17] Merin RG, Bernard JM, Doursout MF, Cohen M, Chelly JE (1991). Comparison of the effects of isoflurane and desflurane on cardiovascular dynamics and regional blood flow in the chronically instrumented dog. Anesthesiology.

[18] Weiskopf RB Holmes MA Eger EI Johnson B H Rampil I J Brown J G, ed Cardiovascular effects of I653 in swine Anesthesiology 1988 69 (3) 303 9 10.1097/00000542-198809000-00003 2nd 3415011 10.1097/00000542-198809000-00003

[19] Pagel PS, Kampine JP, Schmeling WT, Warltier DC (1991). Comparison of the systemic and coronary hemodynamic actions of desflurane, isoflurane, halothane, and enflurane in the chronically instrumented dog. Anesthesiology.

[20] Shanewise JS, Cheung AT, Aronson S, Stewart WJ, Weiss RL, Mark JB (1999). ASE/SCA guidelines for performing a comprehensive intraoperative multiplane transesophageal echocardiography examination: recommendations of the American Society of Echocardiography Council for Intraoperative Echocardiography and the Society of Cardiovascular Anesthesiologists Task Force for Certification in Perioperative Transesophageal Echocardiography. Journal of the American Society of Echocardiography: official publication of the J Am Soc Echocardiogr.

[21] Quasha AL Eger EI Tinker J H, ed Determination and applications of MAC Anesthesiology 1980 53 (4) 315 34 10.1097/00000542-198010000-00008 2nd 6107067 10.1097/00000542-198010000-00008

[22] Eger EI, Johnson B H, Weiskopf R B, Holmes M A, Yasuda N, Targ A (1988). Minimum alveolar concentration of I-653 and isoflurane in pigs: definition of a supramaximal stimulus. Anesth Anal.

[23] Moeser AJ, Blikslager AT, Swanson C (2008). Determination of minimum alveolar concentration of sevoflurane in juvenile swine. Res Vet Sci.

[24] Michard F, Chemla D, Richard C, Wysocki M, Pinsky MR, Lecarpentier Y (1999). Clinical use of respiratory changes in arterial pulse pressure to monitor the hemodynamic effects of PEEP. Am J Respir Crit Care Med.

[25] Hofer CK, Cannesson M (2011). Monitoring fluid responsiveness. Acta Anaesthesiol Taiwan.

[26] Michard F (2005). Changes in arterial pressure during mechanical ventilation. Anesthesiology.

[27] Otsuki DA, Fantoni DT, Holms C, Auler JO (2010). Minimum alveolar concentrations and hemodynamic effects of two different preparations of sevoflurane in pigs. Clinics.

